# Lung Cancer Screening Eligibility and Referral Practices in Texas Organizations Serving People with Substance Use Disorders

**DOI:** 10.3390/cancers15072073

**Published:** 2023-03-30

**Authors:** Maggie Britton, Tzuan A. Chen, Isabel Martinez Leal, Anastasia Rogova, Bryce Kyburz, Teresa Williams, Mayuri Patel, Randa El-Zein, Eric H. Bernicker, Lisa M. Lowenstein, Lorraine R. Reitzel

**Affiliations:** 1 Department of Health Disparities Research, The University of Texas MD Anderson Cancer Center, 1400 Pressler Street, Houston, TX 77030, USA; 2 Department of Psychological, Health & Learning Sciences, University of Houston, 3657 Cullen Blvd. Stephen Power Farish Hall, Houston, TX 77204, USA; 3 HEALTH Research Institute, The University of Houston, 4349 Martin Luther King Blvd., Houston, TX 77204, USA; 4 Integral Care, 1430 Collier St., Austin, TX 78704, USA; 5 Department of State Health Services, Tobacco Prevention and Control Branch, Austin, TX 78714, USA; 6 Houston Methodist Neal Cancer Center, 6445 Main Street, Houston, TX 77030, USA; 7 Department of Health Services Research, The University of Texas MD Anderson Cancer Center, 1400 Pressler Street, Houston, TX 77030, USA

**Keywords:** lung cancer screening, substance use disorder treatment, early detection, cigarette smoking

## Abstract

**Simple Summary:**

People with substance use disorders have extremely elevated rates of smoking and, therefore, are a priority population for lung cancer screening. This paper examines the lung cancer screening practices—determining patients’ eligibility for lung cancer screening and making referrals to screening—of Texas healthcare organizations that provide services to people with substance use disorders. This work demonstrated that few organizations are determining patients’ eligibility and even fewer are making referrals. While not all organizations have the capability to make referrals (i.e., no on-site prescriber), they each have a vital role to play in eligibility determination and patient education. There is a need for researchers to focus intervention and implementation efforts within these organizations to increase capacity and ensure that patients are being navigated to lung cancer screening at multiple touch points across the healthcare continuum.

**Abstract:**

For people at elevated risk for lung cancer, lung cancer screening (LCS) reduces lung cancer mortality. People with non-nicotine substance use disorders (SUDs) have elevated rates of smoking compared with the general population, highlighting them as a priority population for LCS consideration. Although research has shown LCS is underutilized, there is little literature to inform whether organizations that serve individuals with SUDs have existing clinical protocols surrounding LCS. In the current study, we examine the LCS eligibility and referral practices among these organizations. We conducted a statewide needs assessment survey in 2021 to discern how tobacco use was being addressed at Texas organizations that provide treatment or services to individuals with SUDs. Respondents were asked to report on their center’s LCS eligibility and referral practices. The analytic sample consists of 125 respondents who represented 23 federally qualified health centers, 29 global local mental health authorities (LMHAs), 12 substance use treatment programs in LMHAs, and 61 standalone substance use treatment centers. Very few respondents indicated that healthcare providers at their center made referrals to LCS for patients (8.8%); a few respondents indicated that their healthcare providers assessed patients’ eligibility for LCS but did not make referrals (3.2%). Intervention and implementation efforts are needed in these and other SUD healthcare settings to bolster organizational capacity and ensure that patients are being navigated to lung cancer screening at multiple touch points across the care continuum.

## 1. Introduction

The rate of annual lung cancer diagnoses in the United States (US) has been gradually declining over recent decades, now accounting for only 12.3% of new cancer cases [1,2]. Despite this, lung cancer deaths make up 21.4% of all cancer deaths and more people die from lung cancer than any other cancer [2,3]. Lung cancer survival decreases proportionate to disease progression at the time of diagnosis; the average survival for the most common lung cancer (i.e., non-small cell) diagnosed at the localized stage is 64%, 37% at the regional stage, and 8% at the distant stage. Unfortunately, more than half of lung cancers are diagnosed at the distant stage because symptoms do not develop until the disease is more advanced [4]. Thus, to improve survival rates, it is imperative to screen people who are at high risk for lung cancer while they are still asymptomatic.

Smoking cigarettes (hereafter, smoking) is the primary risk factor for lung cancer. Smoking accounts for 90% of all lung cancer diagnoses and for about 80–90% of lung cancer deaths [5,6]. Furthermore, persons who smoke are 15–30 times more likely to be diagnosed with lung cancer compared with persons who do not smoke [5]. In line with this, the United States Preventive Services Task Force (USPSTF) recommends lung cancer screening with low-dose computed tomography for asymptomatic people who meet all the following (high-risk) criteria: (1) aged 50 to 80 years, (2) have a 20 pack/year smoking history, and (3) either currently smoke, or have quit smoking within the past 15 years [6]. To be eligible for lung cancer screening, people must also have no history of prior lung cancer, be in good health, and have no symptoms of lung cancer; if a person develops a health problem that substantially limits their life expectancy or ability to have surgery if lung cancer is found, then they are not a good candidate for lung cancer screening [6]. Low-dose computed tomography is the only recommended lung cancer screening test that has demonstrated utility in supporting early detection, and its use leads to at least a 20% reduction in lung cancer deaths (as compared to chest X-ray) [6,7,8,9].

Although the estimated rate of smoking among the general US population has fallen to a record low of 12.5% [10], the rate of smoking for people with non-nicotine substance use disorders (SUDs) remains unduly high, with some estimates ranging from 65% to over 90% [11,12,13]. Most individuals with SUD who smoke have tobacco use disorder (despite not being medically evaluated for it) [14,15], highlighting them as a priority population for tobacco control policies and practices. State-level policies and community–academic partnerships have pioneered this work within organizations serving people with SUDs [16,17,18,19] but have yet to focus on increasing rates of lung cancer screening in this population. This is a reasonable setting in which to address lung cancer screening because, in the US, 11.3% of adults 50 years and older reported a past year SUD in 2021 [20]. In certain geographical regions, lung cancer screening rates are especially low; for example, despite the national average of 5.7% of eligible adults being screened, in Texas, that number is only 1.9% [21,22]. In the current paper, we examine the lung cancer screening eligibility and referral practices across Texas organizations that serve people with SUDs. 

## 2. Materials and Methods

### 2.1. Participants and Procedure

Data were collected from April–December of 2021 in a statewide needs assessment conducted to ascertain the landscape of tobacco control policies and practices within healthcare organizations providing SUD treatment across Texas. Targeted healthcare centers included (1) federally qualified health centers (FQHCs), which are safety-net agencies that provide low-to-no cost health care (based on a sliding fee scale proportionate to patient income), including behavioral health services [23]; (2) global local mental health authorities (LMHAs), which provide low-cost behavioral health care to patients primarily diagnosed with mental health disorders across Texas (there are 39 global LMHAs and each serves a distinct geographic region) [24]; (3) substance use treatment (SUT) programs within LMHAs, which provide SUD treatment and, although technically they operate under the umbrella of a global LMHA, in practice they operate quite independently; and (4) standalone substance use treatment centers (SUTCs), which provide SUD treatment that is usually focused (e.g., opioid treatment centers) or broad (i.e., any SUD) in scope, as well as centers that provide dual-diagnosis (i.e., mental health and SUD treatment). Centers were identified using publicly available lists and websites [25,26,27], as well as through attendance at professional conferences (e.g., Texas Association of Addiction Professionals, United States Association of Opioid Treatment Providers) [28,29]. Contact information for center representatives was likewise obtained through public websites (e.g., the center’s website or attendance lists from meetings on public record), calling the centers, and through attendance at professional conferences and community treatment provider meetings (e.g., community resource coordination groups) [30].

Data were collected via electronic survey. The survey link was distributed using a multi-pronged recruitment strategy that included direct emailing, postal mailing (when email contact information was not obtained), recruitment at professional conferences and community organization meetings, and distribution via professional organizations’ listservs. The survey cover letter solicited one survey per physical healthcare center location to be completed by an employee most familiar with how tobacco use was being addressed (given the scope of the contracted needs assessment); thus, the appropriate respondent was identified by the center. However, duplicate responses were identified (*n* = 10 respondents representing five physical locations), and a single appropriate respondent was retained by the study team based on respondent job titles (e.g., executive director retained over administrative assistant), the level of completeness of the survey (i.e., completed surveys were retained over incomplete surveys), and information from community treatment collaborators about the most credible source of information (e.g., advised retention of a senior clinical administrator over a certified tobacco treatment specialist). Respondents who completed at least 75% of the survey items were offered a $20 Amazon gift card. This project did not meet the definition of human subjects research under 45 CFR 46.102 (I) per the University of Houston compliance office; therefore, no IRB approval was required.

### 2.2. Measures

#### 2.2.1. Lung Cancer Screening

Respondents were asked whether their organization’s healthcare providers assess for eligibility and/or refer individuals who meet eligibility criteria for lung cancer screening. Respondents indicated either that their organization’s healthcare providers made lung cancer screening referrals, screened only for eligibility but did not make referrals, or indicated that they did neither or did not know.

#### 2.2.2. Employee Role

Respondents were asked whether they were a direct service provider. Direct service providers were defined as employees providing treatment for behavioral health needs (i.e., substance use disorders and/or clinical dependencies) to patients aged 16 or older at the workplace.

#### 2.2.3. Center-Level Characteristics

Respondents reported on the following center-level characteristics: (1) the number of unique annual patients (based on sample distributions, presented as: 50–200; 201–1000; >1000), (2) the number of full-time employees (based on sample distributions, presented as: 1–50; >50), and (3) whether the organization employed someone with prescribing capabilities (i.e., someone who could provide the screening order).

### 2.3. Statistical Analysis

Quantitative data were analyzed using SAS 9.4. Descriptive statistics are used to present findings. Center-level characteristics are presented overall and by each of the four healthcare center types (Table 1). Lung cancer screening practices are presented overall and by each of the four healthcare center types, by employee role, and by prescribing capability (Table 2). Differences were assessed using chi-square tests (*p* < 0.05).

## 3. Results

### 3.1. Analytic Sample

Respondents (*n* = 135 total) were employees of organizations in Texas that provide healthcare to individuals with SUDs, representing FQHCs (*n* = 25, of 57 solicited; ~44% response rate), global LMHAs (*n* = 30, of 39 solicited; ~77%), identified SUT programs in LMHAs (*n* = 14, of 89 solicited; ~16%), and identified SUTCs (*n* = 66, of 458 solicited; ~14%). Overall, 10 respondents failed to complete the questions on lung cancer screening, yielding an analytic sample of 125 respondents who represented 23 FQHCs, 29 global LMHAs, 12 SUT programs in LMHAs, and 61 standalone SUTCs.

### 3.2. Descriptive Statistics

Over half of respondents were direct service providers (*n* = 79; 63.20%), e.g., licensed counselor, psychologist, nurse practitioner, tobacco cessation specialist, recovery specialist, etc. Non-direct service provider (i.e., general employee) roles included chief executive officer, executive director, chief operations officer, clinic manager, program director, etc. Respondents were employed at centers that served a large range of patients each year. Over half of centers had 50 or fewer employees and an employee with prescribing capabilities. There were significant differences between centers on demographic information. LMHAs served many patients, with three-quarters of LMHAs serving over 1000 patients annually. SUTCs, on the other hand, served fewer patients, with nearly all serving between 50 and 1000 patients annually. Accordingly, LMHAs generally employed more people and SUTCs generally employed fewer people. Finally, LMHAs, FQHCs, and SUT programs within LMHAs were more likely to have a professional onsite with prescribing capabilities^2^ compared with SUTCs (see Table 1). 

### 3.3. Main Findings

Overall, very few respondents reported that their healthcare providers made referrals to lung cancer screening. A few additional respondents reported that they assessed patients’ eligibility but did not make referrals. The remaining respondents indicated that their center’s healthcare providers did not assess eligibility or make referrals or that they did not know their center’s procedures.

Lung cancer screening practices were significantly different by center type and prescribing capability. Referrals were more likely to be made at FQHCs and eligibility assessments were more likely to occur at SUTCs. All the centers in which respondents indicated providers were making referrals employed at least one provider with prescribing capabilities (see Table 2).

## 4. Discussion

There is a dire need in the US to increase the number of people receiving lung cancer screening [22]. One strategy to navigate more people toward lung cancer screening is to conduct eligibility screening and make referrals in the places where high-risk people (as defined by the USPSTF) receive health care [6]. People with SUDs have extremely elevated rates of smoking [11,12,13]; therefore, healthcare organizations that provide care to people with SUDs are one location in which to assess lung cancer screening eligibility. We examined the lung cancer screening practices of Texas healthcare organizations that serve people with SUDs.

Unfortunately, very few respondents indicated that healthcare providers at their organization make referrals to lung cancer screening; among the ones that do, FQHCs were most represented. This is reasonable given that referrals to lung cancer screening need to be made by someone with prescribing capabilities, and FQHCs (which have a mission of providing comprehensive health care services to underserved communities) should have such medical professionals (e.g., medical doctors, nurse practitioners, physician assistants) employed (and did, in this sample; 95.45%). One factor limiting certain organizations’ (e.g., standalone SUTCs) referral capacity is that they may not employ professionals who can provide referrals (in the state of Texas, SUTCs often employ licensed chemical dependency counselors, who do not have prescribing capabilities). Accordingly, we found that about half of SUTCs had a professional on site with prescribing capabilities. However, most SUT programs in LMHAs and global LMHAs in our sample employed prescribing medical professionals, but were less likely to report making referrals.

State-level policies and community-academic partnerships have pioneered tobacco control efforts prioritizing people with SUDs, including within Texas where over half of global LMHAs and a handful of SUTCs have implemented comprehensive tobacco-free workplace programs [16,17,18,31,32,33,34,35]. Accordingly, most respondents indicated that their organization mandated that every adult patient be screened for tobacco use; therefore, a natural next step for these organizations would be to assess eligibility for lung cancer screening (using patient tobacco use history) and make referrals (when appropriate and if feasible). Additionally, it is vital to support lung cancer prevention (i.e., smoking cessation) efforts to complement suggested early detection (i.e., screening) efforts in these settings. Patients with SUDs face barriers to tobacco cessation, including limited support from healthcare providers [36]. Therefore, each adult patient that reports tobacco use in SUD care settings should be referred to the state quitline (i.e., the Texas Tobacco Quitline), which offers free support virtually (i.e., over the phone/web) and includes nicotine replacement therapy for qualified callers (e.g., those that receive a direct referral from a healthcare provider) [37]. Future implementation efforts to reduce the research-to-practice translational gap should address bolstering the readiness and capacity of these organizations to couple lung cancer screening practices with their tobacco control programs.

These organizations have an important role to play in increasing the rates of lung cancer screening, especially given that (a) Texas Medicaid covers lung cancer screening in line with the most up-to-date USPSTF recommendation [38], and 21.3% of adults (18+) with a past-year SUD (in the United States) have Medicaid [39], and (b) since the Centers for Medicare and Medicaid Services restriction regarding who can deliver the counseling and shared decision-making visit for lung cancer screening has been removed [40]. Healthcare providers within these organizations can assess whether patients meet the eligibility criteria for lung cancer screening [6], make patients aware of their eligibility (which would need confirmation by a medical professional), deliver the counseling (e.g., smoking cessation intervention) and shared decision-making visit (e.g., risks and benefits of screening, elicitation of values, and elicitation of preferences), and provide documentation of the counseling and shared decision-making visit to their physician, therefore removing barriers to the physician placing the order.

It is important to acknowledge that, in some SUD care settings (e.g., standalone SUTCs, SUT programs within LMHAs), while there may be programs in place (e.g., alumni events) to support long-term abstinence, there is no set expectation that the patient return for an annual health care visit (unlike in an FQHC setting) beyond their time in active treatment (which may last only a few months). Therefore, the role of these SUD care settings may be in addressing initial lung cancer screening eligibility/referral and not in addressing adherence to subsequent annual screenings. However, to facilitate early detection and achieve reductions in lung cancer mortality, it is essential that screening be conducted annually for individuals meeting high risk criteria [6,7].

Another SUD care setting that was not assessed in the present study, but that may be better positioned (vs. SUD treatment settings) to addressing adherence to annual lung cancer screening, is recovery organizations such as Alcoholics Anonymous (AA) or Narcotics Anonymous (NA). Unlike in treatment programs, you do not “graduate” from recovery programs such as AA or NA. Instead, it is encouraged that people return for support as needed and/or especially for anniversary dates (e.g., picking up an annual sobriety chip). For example, in NA, the average length of sobriety is over 11 years and members attend 2–2.5 meetings per week (for NA and AA, respectively). Furthermore, both AA and NA have higher proportions of older adults than SUD care settings; 53% of adults in AA and 40% of adults in NA are >50 years old [41,42]. Although these settings may not have healthcare providers present, there is still an opportunity to provide lung cancer screening education and navigation to services. Future work may additionally consider the role of SUD treatment and recovery organizations not only for lung cancer screening relevant to early detection, but also the role of screening in facilitating better oncologic outcomes and functioning following treatment and in survivorship (i.e., surveillance) [43,44,45,46].

Numerous organizations have developed resources to support service delivery, including templates for assessing lung cancer screening eligibility with a tobacco use assessment [47], as well as tools for delivery of counseling and conducting the shared decision-making visit [48,49,50]. There are also patient educational materials to support patients’ shared decision-making with their doctor that include a video and paper decision aid [33,34,35]. Finally, there are resources available that can support informed decision-making for patients, including rack cards (i.e., brochures) on the risks and benefits of screening [51] and insurance coverage information [52].

There are multiple limitations to this work. First, a single respondent was asked to report on lung cancer screening practices on behalf of the entire center. Therefore, responses could reflect respondents’ own personal knowledge and practices rather than that of the entire center. Future work could assess multiple providers within a single organization to assess agreement among respondents. Second, respondents at centers providing more comprehensive medical and behavioral health services (i.e., FQHCs and LMHAs, respectively) than standalone SUTCs may, despite the directions at the beginning of the survey, have answered the questions about practices for the organization more broadly versus exclusively for patients receiving SUD care. This is a potential limitation to interpretation of the data if, in fact, SUD care is siloed from more general services in these settings, with accompanying differences in protocols for lung cancer screening. Future work should better understand the inner context of treatment settings to help inform implementation strategies for enhancing lung cancer screening. Third, the lung cancer screening question was embedded into a longer survey where the primary purpose was to assess tobacco use knowledge and practices. Accordingly, the lung cancer screening question was positioned at the end of the survey, and 10 respondents did not make it to this item. Future research to address the stated limitations should assess the extent to which executive leadership of these organizations report that lung cancer screening is expected in patient interactions, and whether healthcare professionals within an organization report consistent provision of these services [53,54]. Fourth, the data were collected during the ongoing COVID-19 pandemic. It is unclear whether more imminent health conditions took priority over preventive screenings. Furthermore, the pandemic resulted in dramatic staffing shortages, which may have affected centers’ ability to conduct lung cancer screenings. Future work to attempt replication of these findings is appropriate. Fifth, we did not assess patient composition characteristics that may have been interesting to contextualize the results, such as age, insurance status, and/or race and ethnicity. Sixth, the low response rate for most organization types and resulting sample size may affect the generalizability of the results.

Despite the limitations to the work, this is the first report that we are aware of that focuses on lung cancer screening practices within organizations providing SUD care and reveals an opportunity to leverage implementation science to build organizational and healthcare provider capacity to deliver these services and ultimately increase the number of patients being screened for lung cancer in the state of Texas. Implementation science frameworks (e.g., the Exploration, Preparation, Implementation, Sustainment Framework [EPIS]; the Consolidated Framework for Implementation Research [CFIR], etc.) use theory- and data-driven approaches to prepare for and engage in the implementation process (i.e., through identification of setting-specific barriers and facilitators) [55,56]. For instance, both the EPIS and CFIR frameworks guide identification of inner (e.g., whether a provider with referral capabilities is employed therein) and outer (e.g., patient characteristics, such as smoking rate, age, etc.) organizational characteristics that are associated with effective implementation. Ultimately, leveraging these frameworks has the potential to promote integration of lung cancer screening services (e.g., determining eligibility, making referrals, education efforts, etc.) into the clinical workflow of SUD care organizations and increase the number of people informed about and engaging in lung cancer screening in the state of Texas.

## 5. Conclusions

In a state where less than 2% of the eligible population is currently being screened for lung cancer, no action is too small to work toward increasing this rate. Increasing the reach of lung cancer screening within Texas is particularly important given that the state presents with above average rates of new cases, a below average survival rate, and is in the bottom tier for treatment provision according to the American Lung Association’s 2021 State of Lung Cancer report [21]. A non-exhaustive list of barriers to provision of lung cancer screening in healthcare settings include a lack of resources and services coverage, time constraints for providers, a lack of knowledge by providers, and a lack of awareness or buy-in from patients [57,58]. Implementation science efforts can address these barriers and develop lung cancer screening programs within these organizations [59,60]. This is particularly feasible in healthcare settings with existing protocols and practices for conducting tobacco use screenings and delivering tobacco use disorder interventions and follow-up care. Furthermore, by discussing lung cancer screening (even if referrals cannot be made) at multiple touch points in a patient’s health care continuum, health care providers can promote awareness of, and increase interest in, screening.

## Figures and Tables

**Table 1:** Center-level characteristics (n = 125 healthcare organizations).

		Center Type					
	**Total** **% [*n*]**	FQHC	SUT Program in LMHA	Global LMHA	SUTC	**X^2^**	***p*-Value**
**# of unique patients/yearly**						**48.2194**	**<0.0001**
50–200	**30.33 (37)**	38.10 (8)	33.33 (4)	3.57 (1)	39.34 (24)		
201–1000	**40.98 (50)**	23.81 (5)	50.00 (6)	21.43 (6)	54.10 (33)		
>1000	**28.69 (35)**	38.10 (8)	16.67 (2)	75.00 (21)	6.56 (4)		
**# of full-time employees**						**51.8412**	**<0.0001**
1–50	**60.80 (76)**	56.52 (13)	75.00 (9)	6.90 (2)	85.25 (52)		
>50	**39.20 (49)**	43.48 (10)	25.00 (3)	93.10 (27)	14.75 (9)		
**Has >/= 1 professional on site with prescribing capabilities**						**27.7692**	**<0.0001**
Yes	**75.00 (87)**	95.45 (21)	91.67 (11)	96.30 (26)	52.73 (29)		
No	**25.00 (29)**	4.55 (1)	8.33 (1)	3.70 (1)	47.27 (26)		

Note. FQHC = federally qualified health center; SUT = substance use treatment; LMHA = local mental health authority; SUTC = substance use treatment center; Nine respondents did not answer the question about prescribing capabilities leaving 7.20% missing data on this item. Missing data are excluded from calculations; therefore, percentages in the table reflect valid percentages.

**Table 2:** Lung Cancer Screening Practices by Center Type, Employee Role, and Prescribing Capability (n = 125 healthcare organizations).

	**Center Type**					**X^2^**	***p*-Value**
	**Total** **% [*n*]**	FQHC	SUT Program in LMHA	Global LMHA	SUTC		
**Lung Cancer Screening Practices**						**33.6975**	**<0.0001**
Completes LDCT referrals	**8.80 (11)**	39.13 (9)	0 (0)	3.45 (1)	1.64 (1)		
Eligibility determination only	**3.20 (4)**	0 (0)	0 (0)	3.45 (1)	4.92 (3)		
Neither/unknown	**88.00 (110)**	60.87 (14)	100 (12)	93.10 (27)	93.44 (57)		
	**Respondents’ Employee Role**					**X^2^**	***p*** **-value**
	**Total**	Direct Service Provider		General Employee			
**Lung Cancer Screening Practices**						**3.1044**	**0.2002**
Completes LDCT referrals	**8.80 (11)**	7.59 (6)		10.87 (5)			
Eligibility determination only	**3.20 (4)**	1.27 (1)		6.52 (3)			
Neither/unknown	**88.00 (110)**	91.14 (72)		82.61 (38)			
	**Center-level Prescribing Capability**					**X^2^**	***p*** **-value**
	**Total**	Has >/= 1 professional on site with prescribing capabilities		No prescribers on site			
**Lung Cancer Screening Practices**						**9.0297**	**0.0111**
Completes LDCT referrals	**9.48 (11)**	12.64 (11)		0 (0)			
Eligibility determination only	**3.45 (4)**	1.15 (1)		10.34 (3)			
Neither/unknown	**87.07 (101)**	86.21 (75)		89.66 (26)			

Note. FQHC = federally qualified health center; SUT = substance use treatment; LMHA = local mental health authority; SUTC = substance use treatment center; LDCT = low-dose computed tomography; Nine respondents did not answer the question about prescribing capabilities leaving 7.20% missing data on this item. Missing data are excluded from calculations; therefore, percentages in the table reflect valid percentages.

## Data Availability

The data presented in this study are available upon request from the corresponding author. The data are not publicly available due to funder restrictions and because outcome papers are still being reported from the dataset.

## References

[1] National Cancer Institute Cancer Stat Facts: Lung and Bronchus Cancer. https://seer.cancer.gov/statfacts/html/lungb.html.

[2] American Cancer Society Cancer Facts & Figures 2021. https://www.cancer.org/content/dam/cancer-org/research/cancer-facts-and-statistics/annual-cancer-facts-and-figures/2021/cancer-facts-and-figures-2021.pdf.

[3] Shibuya K., Mathers C.D., Boschi-Pinto C., Lopez A.D., Murray C.J. (2002). Global and Regional Estimates of Cancer Mortality and Incidence by Site: II. Results for the Global Burden of Disease 2000. BMC Cancer.

[4] American Cancer Society Tests for Lung Cancer. https://www.cancer.org/cancer/lung-cancer/detection-diagnosis-staging/how-diagnosed.html.

[5] Center for Disease Control and Prevention What Are the Risk Factors for Lung Cancer?. https://www.cdc.gov/cancer/lung/basic_info/risk_factors.htm.

[6] Moyer V.A. (2014). Screening for Lung Cancer: U.S. Preventive Services Task Force Recommendation Statement. Ann. Intern Med..

[7] Aberle D.R., Adams A.M., Berg C.D., Black W.C., Clapp J.D., Fagerstrom R.M., Gareen I.F., Gatsonis C., Marcus P.M., Sicks J.D. (2011). Reduced Lung-Cancer Mortality with Low-Dose Computed Tomographic Screening-The National Lung Screening Trial Research Team. N. Engl. J. Med..

[8] Pastorino U., Silva M., Sestini S., Sabia F., Boeri M., Cantarutti A., Sverzellati N., Sozzi G., Corrao G., Marchianò A. (2019). Prolonged Lung Cancer Screening Reduced 10-Year Mortality in the MILD Trial: New Confirmation of Lung Cancer Screening Efficacy. Ann. Oncol..

[9] De Koning H.J., van der Aalst C.M., de Jong P.A., Scholten E.T., Nackaerts K., Heuvelmans M.A., Lammers J.-W.J., Weenink C., Yousaf-Khan U., Horeweg N. (2020). Reduced Lung-Cancer Mortality with Volume CT Screening in a Randomized Trial. N. Engl. J. Med..

[10] Centers for Disease Control Burden of Cigarette Use in the U.S.. https://www.cdc.gov/tobacco/campaign/tips/resources/data/cigarette-smoking-in-united-states.html#:~:text=In%202020%2C%20an%20estimated%2012.5,every%20day%20or%20some%20days.

[11] Guydish J., Passalacqua E., Tajima B., Chan M., Chun J., Bostrom A. (2011). Smoking Prevalence in Addiction Treatment: A Review. Nicotine Tob. Res..

[12] Hunt J.J., Gajewski B.J., Jiang Y., Cupertino A.P., Richter K.P. (2013). Capacity of US Drug Treatment Facilities to Provide Evidence-Based Tobacco Treatment. Am. J. Public Health.

[13] Baca C.T., Yahne C.E. (2009). Smoking Cessation during Substance Abuse Treatment: What You Need to Know. J. Subst. Abus. Treat.

[14] Baker T.B., Breslau N., Covey L., Shiffman S. (2012). DSM Criteria for Tobacco Use Disorder and Tobacco Withdrawal: A Critique and Proposed Revisions for DSM-5. Addiction.

[15] American Academy of Addiction Psychiatry (2015). Nicotine Dependence.

[16] Correa-Fernández V., Wilson W.T., Kyburz B., O’Connor D.P., Stacey T., Williams T., Lam C.Y., Reitzel L.R. (2019). Evaluation of the Taking Texas Tobacco Free Workplace Program within Behavioral Health Centers. Transl. Behav. Med..

[17] Le K., Correa-Fernández V., Martinez Leal I., Kyburz B., Chen T.-A., Barrientos D., Saenz E., Williams T., O’Connor D.P., Obasi E.M. (2020). Tobacco-Free Workplace Program at a Substance Use Treatment Center. Am. J. Health Behav..

[18] Martinez Leal I., Chen T.-A., Correa-Fernández V., Le K., O’Connor D.P., Kyburz B., Wilson W.T., Williams T., Reitzel L.R. (2020). Adapting and Evaluating Implementation of a Tobacco-Free Workplace Program in Behavioral Health Centers. Am. J. Health Behav..

[19] Marshall L.L., Kuiper N.M., Lavinghouze S.R. (2015). Strategies to Support Tobacco Cessation and Tobacco-Free Environments in Mental Health and Substance Abuse Facilities. Prev. Chronic Dis..

[20] Substance Abuse and Mental Health Services Administration (2021). National Survey on Drug Use and Health (NSDUH): Detailed Tables, Table 5.4B–Drug Use Disorder, Alcohol Use Disorder, and Substance Use Disorder in Past Year: Among People Aged 12 or Older; by Detailed Age Category, Percentages.

[21] American Lung Association State of Lung Cancer: Texas. https://www.lung.org/research/state-of-lung-cancer/states/texas.

[22] American Lung Association Lung Cancer Key Findings. https://www.lung.org/research/state-of-lung-cancer/key-findings.

[23] Health Resources & Services Administration Health Center Program Award Recipients. https://www.hrsa.gov/opa/eligibility-and-registration/health-centers/fqhc.

[24] Texas Health and Human Services Local Mental Health Authorities. https://www.hhs.texas.gov/providers/behavioral-health-services-providers/local-mental-health-authorities.

[25] Health Resources & Services Administration Find a Health Center. https://findahealthcenter.hrsa.gov/.

[26] Texas Health and Human Services Chemical Dependency Treatment Facilities. https://www.hhs.texas.gov/providers/health-care-facilities-regulation/chemical-dependency-treatment-facilities.

[27] Texas Health and Human Services Find Your Local Mental Health or Behavioral Health Authority. https://www.hhs.texas.gov/services/mental-health-substance-use/mental-health-substance-use-resources/find-your-local-mental-health-or-behavioral-health-authority.

[28] Texas Association of Addiction Professionals. https://www.taap.org/.

[29] The United States Association of Opioid Treatment Providers. http://www.usaotp.org/home.html.

[30] Community Resource Coordination Groups. https://crcg.hhs.texas.gov/.

[31] Taing M., Nitturi V., Chen T.A., Kyburz B., Martinez Leal I., Correa-Fernández V., Obasi E.M., Williams T., Casey K., O’Connor D.P. (2021). Implementation and Outcomes of a Comprehensive Tobacco Free Workplace Program in Opioid Treatment Centers. Int. J. Env. Res. Public Health.

[32] Garey L., Neighbors C., Martinez Leal I., Lam C.Y., Wilson W.T., Kyburz B., Stacey T., Correa-Fernández V., Williams T., Zvolensky M.J. (2019). Tobacco-Related Knowledge Following a Comprehensive Tobacco-Free Workplace Program within Behavioral Health Facilities: Identifying Organizational Moderators. Patient Educ. Couns..

[33] Taing M., Kyburz B., Martinez Leal I., Le K., Chen T.-A., Correa-Fernandez V., Williams T., O’Connor D.P., Obasi E.M., Casey K. (2020). Clinician Training in the Adaptation of a Comprehensive Tobacco-Free Workplace Program in Agencies Serving the Homeless and Vulnerably Housed. Int. J. Environ. Res. Public Health.

[34] Nitturi V., Chen T.-A., Kyburz B., Martinez Leal I., Correa-Fernandez V., O’Connor D.P., Williams T., Garey L., Stacey T., Wilson W.T. (2021). Organizational Characteristics and Readiness for Tobacco-Free Workplace Program Implementation Moderates Changes in Clinician’s Delivery of Smoking Interventions within Behavioral Health Treatment Clinics. Nicotine Tob. Res..

[35] Correa-Fernández V., Wilson W.T., Shedrick D.A., Kyburz B., Samaha H.L., Stacey T., Williams T., Lam C.Y., Reitzel L.R. (2017). Implementation of a Tobacco-Free Workplace Program at a Local Mental Health Authority. Transl. Behav. Med..

[36] Twyman L., Bonevski B., Paul C., Bryant J. (2014). Perceived Barriers to Smoking Cessation in Selected Vulnerable Groups: A Systematic Review of the Qualitative and Quantitative Literature. BMJ Open.

[37] Texas Tobacco Quitline. https://www.yesquit.org/.

[38] American Lung Association State Lung Cancer Screening Coverage Toolkit. https://www.lung.org/lung-health-diseases/lung-disease-lookup/lung-cancer/screening-resources/state-lung-cancer-screening.

[39] Substance Abuse and Mental Health Services Administration (2021). National Survey on Drug Use and Health (NSDUH): Detailed Tables, Table 5.10B–Substance Use Disorder in Past Year: Among People Aged 12 or Older; by Age Group and Geographic and Socioeconomic Characteristics, Percentages.

[40] Centers for Medicare & Medicaid Services Screening for Lung Cancer with Low Dose Computed Tomography (LDCT). https://www.cms.gov/medicare-coverage-database/view/ncacal-decision-memo.aspx?proposed=N&ncaid=304.

[41] Narcotics Anonymous (2018). 2018 Narcotics Anonymous Membership Survey. https://www.na.org/admin/include/spaw2/uploads/pdf/pr/2301_MS_2018.pdf.

[42] Alcoholics Anonymous (2014). Alcoholics Anonymous 2014 Membership Survey. https://www.aa.org/sites/default/files/literature/assets/p-48_membershipsurvey.pdf.

[43] Schneider B.J., Ismaila N., Altorki N. (2020). Lung Cancer Surveillance After Definitive Curative-Intent Therapy: ASCO Guideline Summary. JCO Oncol. Pract..

[44] Colt H.G., Murgu S.D., Korst R.J., Slatore C.G., Unger M., Quadrelli S. (2013). Follow-up and Surveillance of the Patient with Lung Cancer After Curative-Intent Therapy. Chest.

[45] Jaklitsch M.T., Jacobson F.L., Austin J.H.M., Field J.K., Jett J.R., Keshavjee S., MacMahon H., Mulshine J.L., Munden R.F., Salgia R. (2012). The American Association for Thoracic Surgery Guidelines for Lung Cancer Screening Using Low-Dose Computed Tomography Scans for Lung Cancer Survivors and Other High-Risk Groups. J. Thorac. Cardiovasc. Surg..

[46] Alberts W.M. (2007). Follow up and Surveillance of the Patient with Lung Cancer: What Do You Do after Surgery?. Respirology.

[47] Taking Texas Tobacco Free Provider Resources. https://www.takingtexastobaccofree.com/provider-materials.

[48] Volk R.J., Lowenstein L.M., Leal V.B., Escoto K.H., Cantor S.B., Munden R.F., Rabius V.A., Bailey L., Cinciripini P.M., Lin H. (2020). Effect of a Patient Decision Aid on Lung Cancer Screening Decision-Making by Persons Who Smoke. JAMA Netw. Open.

[49] Patient-Centered Outcomes Research Institute (PCORI) Implementing Patient Decision Support for Lung Cancer Screening through Tobacco Quitlines. https://www.pcori.org/research-results/2019/implementing-patient-decision-support-lung-cancer-screening-through-tobacco-quitlines.

[50] Project CONNECT Lung Cancer Screening Support. https://lungscreen.health/.

[51] Taking Texas Tobacco Free Download Center. https://www.takingtexastobaccofree.com/download-center-home.

[52] American Lung Association Lung Cancer Screening Insurance Checklist. https://www.lung.org/lung-health-diseases/lung-disease-lookup/lung-cancer/screening-resources/insurance-checklist.

[53] Reitzel L.R. (2022). DP20-2001 Behavioral Health Project (HHS000961900001).

[54] Reitzel L. Taking Texas Tobacco Free among Substance Users within Community-Based Healthcare Settings in Rural and Medically Underserved Areas Across Texas (PP210003). Cancer Prevention and Research Institute of Texas 2021. https://www.cprit.state.tx.us/grants-funded/grants/pp210003.

[55] Moullin J.C., Dickson K.S., Stadnick N.A., Rabin B., Aarons G.A. (2019). Systematic Review of the Exploration, Preparation, Implementation, Sustainment (EPIS) Framework. Implement. Sci..

[56] Damschroder L.J., Reardon C.M., Widerquist M.A.O., Lowery J. (2022). The Updated Consolidated Framework for Implementation Research Based on User Feedback. Implement. Sci..

[57] Wang G.X., Baggett T.P., Pandharipande P.V., Park E.R., Percac-Lima S., Shepard J.-A.O., Fintelmann F.J., Flores E.J. (2019). Barriers to Lung Cancer Screening Engagement from the Patient and Provider Perspective. Radiology.

[58] Mejia M.C., Zoorob R., Gonzalez S., Mosqueda M., Levine R. (2021). Key Informants’ Perspectives on Implementing a Comprehensive Lung Cancer Screening Program in a Safety Net Healthcare System: Leadership, Successes, and Barriers. J. Cancer Educ..

[59] Zeliadt S.B., Hoffman R.M., Birkby G., Eberth J.M., Brenner A.T., Reuland D.S., Flocke S.A. (2018). Challenges Implementing Lung Cancer Screening in Federally Qualified Health Centers. Am. J. Prev. Med..

[60] Watson L., Cotter M.M., Shafer S., Neloms K., Smith R.A., Sharpe K. (2021). Implementation of a Lung Cancer Screening Program in Two Federally Qualified Health Centers. Public Health Rep..

